# Baseline serum angiopoietin-2 and VEGF levels predict the deterioration of the liver functional reserve during lenvatinib treatment for hepatocellular carcinoma

**DOI:** 10.1371/journal.pone.0247728

**Published:** 2021-03-01

**Authors:** Taku Shigesawa, Goki Suda, Megumi Kimura, Osamu Maehara, Yoshimasa Tokuchi, Akinori Kubo, Ren Yamada, Ken Furuya, Masaru Baba, Takashi Kitagataya, Kazuharu Suzuki, Masatsugu Ohara, Naoki Kawagishi, Masato Nakai, Takuya Sho, Mitsuteru Natsuizaka, Kenichi Morikawa, Koji Ogawa, Naoya Sakamoto

**Affiliations:** 1 Department of Gastroenterology and Hepatology, Graduate School of Medicine, Hokkaido University, Sapporo, Japan; 2 Department of Gastroenterology and Hepatology, Japan Community Health Care Organization (JCHO) Hokkaido Hospital, Hokkaido, Japan; Nihon University School of Medicine, JAPAN

## Abstract

A deteriorated liver functional reserve during systemic therapy for unresectable hepatocellular carcinoma (HCC) causes poor patient outcomes. We aimed to identify predictive factors associated with the deterioration of Child-Pugh score at 8 weeks after lenvatinib initiation. Patients with adequate clinical data and baseline preserved serum samples available were included. Baseline fibroblast growth factor (FGF)19 and 21, angiopoietin (ANG)2, and vascular endothelial growth factor (VEGF) levels were evaluated. Thirty-seven patients were included, and 6, 15, 14, and 2 experienced complete response, partial response, stable disease, and progressive disease, respectively. Twenty-four (65%) and 13 (35%) patients showed a maintained/improved and deteriorated Child-Pugh-score, respectively. While baseline clinical data, treatment response, and laboratory data were similar between these two patient groups, baseline ANG2 and VEGF levels were significantly higher (*P* = 0.0017) and lower (*P* = 0.0231), respectively, in patients with deteriorated Child-Pugh score than in those without. Based on receiver operating characteristic curve analysis, cut-off values for ANG2 and VEGF were found to be 3,108 pg/mL and 514.9 pg/mL, respectively. Among patients with low VEGF and high ANG2, 89% (8/9) exhibited a deteriorated Child-Pugh score, whereas none of the patients (0/9) with high VEGF and low ANG2 did. The deterioration of the Child-Pugh score in patients with unresectable HCC who are treated with lenvatinib may be predictable based on combined baseline serum ANG2 and VEGF levels.

## Introduction

Hepatocellular carcinoma (HCC) is one of the major causes of cancer-related death worldwide [1]. Novel therapeutic options and proper treatment strategies for patients with advanced HCC are crucially needed. As various clinical trials on novel systemic therapies for unresectable HCC could not demonstrate significant efficacy of multikinase inhibitors, including sunitinib, brivanib, and linifanib, or even report non-inferiority compared to sorafenib [2–4], systemic therapy is limited to sorafenib alone [5]. This limited therapeutic option might contribute to the poor prognosis of patients with advanced HCC. Quite recently, another multikinase inhibitor, regorafenib [6], and a monoclonal antibody targeting vascular endothelial growth factor receptor (VEGFR) 2, ramucirumab [7], have been approved as second-line systemic therapies, whereas the multikinase inhibitor lenvatinib [8] has been approved as first-line systemic therapy for patients with advanced HCC. More recently, combination therapy with the VEGF inhibitor bevacizumab and programmed death ligand 1 (PD-L1) inhibitor atezolizumab resulted in better overall survival (OS) and progression-free survival (PFS) than sorafenib treatment in patients with unresectable HCC [9]. Therefore, for patients with unresectable HCC, sequential therapy consisting of these novel therapies is currently available. However, most of these patients have a deteriorated liver functional reserve due to chronic liver disease, such as hepatitis C or B virus infection or nonalcoholic steatohepatitis, affecting their prognosis [10] and limiting their therapeutic options.

Lenvatinib, which targets VEGFR1–3, fibroblast growth factor receptors 1–4, platelet-derived growth factor receptor-α, c-Kit, and rearranged during transfection proto-oncogene [11, 12], demonstrated non-inferiority to sorafenib in terms of OS in unresectable HCC [8]. The efficacy and safety of lenvatinib in patients with unresectable HCC have been demonstrated in a real-world setting [13–17]. However, some patients experienced deterioration of the liver functional reserve during lenvatinib treatment for HCC. Recently, Terashima et al. reported that maintenance of the liver functional reserve during tyrosine kinase inhibitor (TKI) therapy for advanced HCC contributed to better OS and PFS [18]. However, predictive factors for deteriorating liver functional reserves during TKI therapy have not been fully elucidated.

Yang et al. reported that normal hepatic sinusoidal microvessel density was reduced by VEGF-specific blockade [19]. A reduced liver blood flow may cause liver functional reserve deterioration; thus, we hypothesized that baseline serum growth factors, including VEGF, fibroblast growth factors (FGFs), and angiopoietin (ANG), might be involved in liver functional reserve deterioration during TKI therapy. Therefore, we evaluated their association with deterioration of the liver functional reserve at 8 weeks after lenvatinib initiation for unresectable HCC.

## Materials and methods

### Patients and study design

We screened patients with unresectable HCC who were treated with lenvatinib at Hokkaido University Hospital and related hospitals between April 2018 and January 2020. Patients were included if they were treated with lenvatinib, were followed for more than 2 months, underwent dynamic computed tomography (CT) at baseline and every 2 to 3 months for evaluation of the treatment response, and had baseline preserved serum samples for the evaluation of serum growth factors as biomarkers and adequate clinical data available. Patients were excluded if they were followed for less than 2 months, received lenvatinib in conjunction with other treatments, had insufficient clinical data or no baseline preserved serum samples available, or lacked proper treatment response evaluation by dynamic CT.

Baseline clinical data, including sex; age; etiology of HCC; laboratory data, including alpha-fetoprotein (AFP) and des-gamma-carboxyprothrombin; Barcelona Clinic Liver Cancer (BCLC) stage; Child-Pugh score; and serum levels of FGF19, FGF21, ANG2, and VEGF, were collected. The patients were evaluated using laboratory tests and physical examination every 2 weeks after lenvatinib initiation. The treatment response was evaluated based on enhanced CT every 2 to 3 months after lenvatinib initiation.

This study was approved by the ethics committee of Hokkaido University Hospital (approval number: 017–0521). All patient’s data were fully anonymized, and were obtained from Hokkaido University Hospital and JCHO Hokkaido Hospital. The date range during which patients’ medical records were accessed, was April 2018-January 2020. All patients provided written informed consent to participate in the study and were provided the option to decline participation. This study conformed to the ethics guidelines of the Declaration of Helsinki.

### Evaluation of treatment response and changes in liver functional reserves

Changes in liver functional reserves were evaluated as changes in the Child-Pugh score between baseline and 8 weeks post treatment initiation, since we evaluated most patients’ treatment response using an enhanced CT at 8 weeks post treatment and decided to either continue or discontinue lenvatinib treatment. Treatment responses to lenvatinib were evaluated every 2 to 3 months after lenvatinib initiation using the modified Response Evaluation Criteria in Solid Tumors criteria, based on dynamic CT [20]. The best response during the treatment was defined as the treatment response.

### Analysis of baseline serum biomarkers

Serum VEGF, ANG2, FGF19, and FGF21 levels were evaluated using commercial enzyme-linked immunosorbent assays (VEGF, ANG2, and FGF19: R&D Systems, Minneapolis, MN, USA; FGF21: Merck Millipore, Darmstadt, Germany) according to the manufacturers’ protocols [14].

### Treatment protocol

Lenvatinib was administered orally once daily at a dose of 8 or 12 mg for patients who weighed <60 or ≥60 kg, respectively. Lenvatinib was discontinued if disease progression or unacceptable adverse events were observed. The lenvatinib dose was adjusted by the attending physician based on adverse events and tolerability.

### Statistical analysis

Categorical variables were analyzed using the chi-squared and Fisher’s exact tests, and continuous variables were analyzed using the paired Mann–Whitney U-test. The relationship between serum ANG2 levels and VEGF levels was analyzed using Spearman’s rank correlation. To compare differences between 3 or more populations, we used one-way analysis of variance followed by Tukey’s test. A multivariate logistic regression analysis with stepwise forward selection was conducted with variables identified as significant at *P* ≤ 0.05 in univariate analyses (baseline serum ANG2 and VEGF levels). Optimal cut-off values of baseline serum ANG2 and VEGF levels were determined based on receiver operating characteristic (ROC) curves by maximizing the Youden index. *P* < 0.05 was considered statistically significant. Statistical analyses were performed using Prism 7.03 (GraphPad Software, La Jolla, CA) software packages.

## Results

### Patient enrollment and baseline characteristics

We screened a total of 59 patients with unresectable HCC who were treated with lenvatinib at Hokkaido University Hospital and related hospitals between April 2018 and January 2020. Of these patients, 22 were excluded from analysis; 6 patients were treated with other anticancer therapies, including transarterial chemoembolization, 1 patient was lost to follow-up within 2 months, and for 15 patients, there was insufficient clinical data and/or lack of baseline preserved serum. Finally, 37 patients with unresectable HCC were included in this study (S1 Fig).

Table 1 shows the baseline patient characteristics. The median patient age was 71 years (range, 54–88 years), and 35 (94.6%) patients were males. Fifteen, 12, and 10 patients had Child-Pugh scores of 5, 6, and ≥7, respectively. No patients had initiated the direct acting antivirals for hepatitis C or nucleoside analog for hepatitis B during the course of lenvatinib treatment or within 3 months prior. Most patients had BCLC stage C (27/37, 73.0%), while 11 (29.7%) had extrahepatic metastases. The median serum AFP level was 20.9 IU/mL (range, 1.3–449,909 IU/mL). Table 2 shows the best treatment response to lenvatinib for HCC in this study. Six, 15, 14, and 2 patients showed complete response, partial response, stable disease, and partial disease, respectively.

**Table 1:** Baseline patient characteristics.

Variable	
**Age (years), median (range)**	71 (54–88)
**Sex (male/female)**	35/2
**Etiology, n (%)**	
HBV	12 (32.4%)
HCV	6 (16.2%)
NBNC	19 (51.3%)
**Vascular invasion, n (%)**	10 (27.0%)
**Extrahepatic extension, n (%)**	11 (29.7%)
**BCLC stage, n (%)**	
B	11 (29.7%)
C	26 (70.0%)
**Child-Pugh class, n (%)**	
A	27 (73.0%)
B	10 (27.0%)
**Child-Pugh score, n (%)**	
5	15 (40.5%)
6	12 (32.4%)
≥7	10 (27.0%)
**Biochemical analysis, median (range)**	
Platelet, *104/μL	16.1 (4.4–51.7)
AST, IU/L	34 (17–181)
ALT, IU/L	23 (11–96)
AFP, ng/mL	20.9 (1.3–449909)
PIVKA-II, mAU/mL	408.5 (13–195319)

HBV: hepatitis B virus, HCV: hepatitis C virus, NBNC: non-B non-C, BCLC: Barcelona Clinic Liver Cancer, AST: aspartate aminotransferase, ALT: alanine aminotransferase, AFP: alpha-fetoprotein, PIVKA-II: protein induced by vitamin K absence or antagonist-II.

**Table 2:** Treatment response comparison between patients with or without deterioration of Child-Pugh scores during treatment with lenvatinib for HCC.

Without deterioration of Child-Pugh score (n = 24)				With deterioration of Child-Pugh score (n = 13)			
PD	SD	PR	CR	PD	SD	PR	CR
1	7	11	5	1	7	4	1
(4.2%)	(29.1%)	(45.8%)	(20.8%)	(7.7%)	(53.8%)	(30.7%)	(7.7%)

*P* = 0.4039 (Without deterioration of Child-Pugh score vs. With deterioration of Child-Pugh score).

PD: progressive disease, SD: stable disease, PR: partial response, CR: complete response.

### Comparison of patient characteristics between patients with and without deterioration of Child-Pugh score at 8 weeks after lenvatinib initiation

As shown in Table 2, 24 (65%) and 13 (35%) patients exhibited a maintained/improved and deterioration of Child-Pugh score, respectively, at 8 weeks post lenvatinib initiation. The most common deterioration factor in the Child-Pugh score was the serum albumin level (S2 Fig). We compared these two patient groups. As shown in Table 3, the etiology of liver disease, sex, age, BCLC stage, Child-Pugh class, and tumor markers were similar between these patient groups. Importantly, the rate of best treatment response was similar between patients with or without deterioration of Child-Pugh score at 8 weeks post lenvatinib initiation (*P* = 0.4039). Thus, even in patients with an objective response, 23.8% (5/21) experienced deterioration of liver function.

**Table 3:** Comparison of patient characteristics between patients with and without deterioration of Child-Pugh score at 8 weeks after lenvatinib initiation.

	Without deterioration (n = 24)	With deterioration (n = 13)	*P* value
**Baseline characteristics**			
**Age (years), median (range)**	72 (55–88)	68 (54–83)	0.138
**Sex (male/female)**	23/1	12/1	>0.999
**Etiology, n (%)**			0.703
HBV	8 (33.3%)	4 (30.8%)	
HCV	3 (12.5%)	3 (23.1%)	
NBNC	13 (54.2%)	6 (46.2%)	
**Vascular invasion, n (%)**	6 (25.0%)	4 (30.8%)	0.715
**Extrahepatic extension, n (%)**	7 (29.2%)	4 (30.8%)	>0.999
**BCLC stage, n (%)**			>0.999
B	7 (29.2%)	4 (30.8%)	
C	17 (70.8%)	9 (69.2%)	
**Child-Pugh class, n (%)**			0.715
A	18 (75.0%)	9 (69.2%)	
B	6 (25.0%)	4 (30.8%)	
**Child-Pugh score, n (%)**			0.165
5	12 (80%)	3 (20%)	
≥6	12 (54.5%)	10 (45.5%)	
**Relative dose intensity (%)**	75.0 (18.8–100)	83.3 (45.8–100)	0.179
**Biochemical analysis, median (range)**			
Platelets −(*10^4^/μL)	18.1 (7.5–51.7)	16.0 (4.4–25.6)	0.081
AST, IU/L	32 (17–72)	38 (21–181)	0.071
ALT, IU/L	23.5 (11–60)	23 (23–96)	0.874
AFP, ng/mL	7 (1.3–449909)	97.5 (5.0–19394.3)	0.394
PIVKA-II, mAU/mL	408.5 (14–29576)	238 (13–195319)	0.33

HBV: hepatitis B virus, HCV: hepatitis C virus, NBNC: non-B non-C, BCLC: Barcelona Clinic Liver Cancer, AST: aspartate aminotransferase, ALT: alanine aminotransferase, AFP: alpha-fetoprotein, PIVKA-II: protein induced by vitamin K absence or antagonist-II, FGF: fibroblast growth factor, ANG2: angiopoietin 2, VEGF: vascular endothelial growth factor.

### Relationship between baseline serum growth factors and changes in the Child-Pugh score

**Fig 1:**
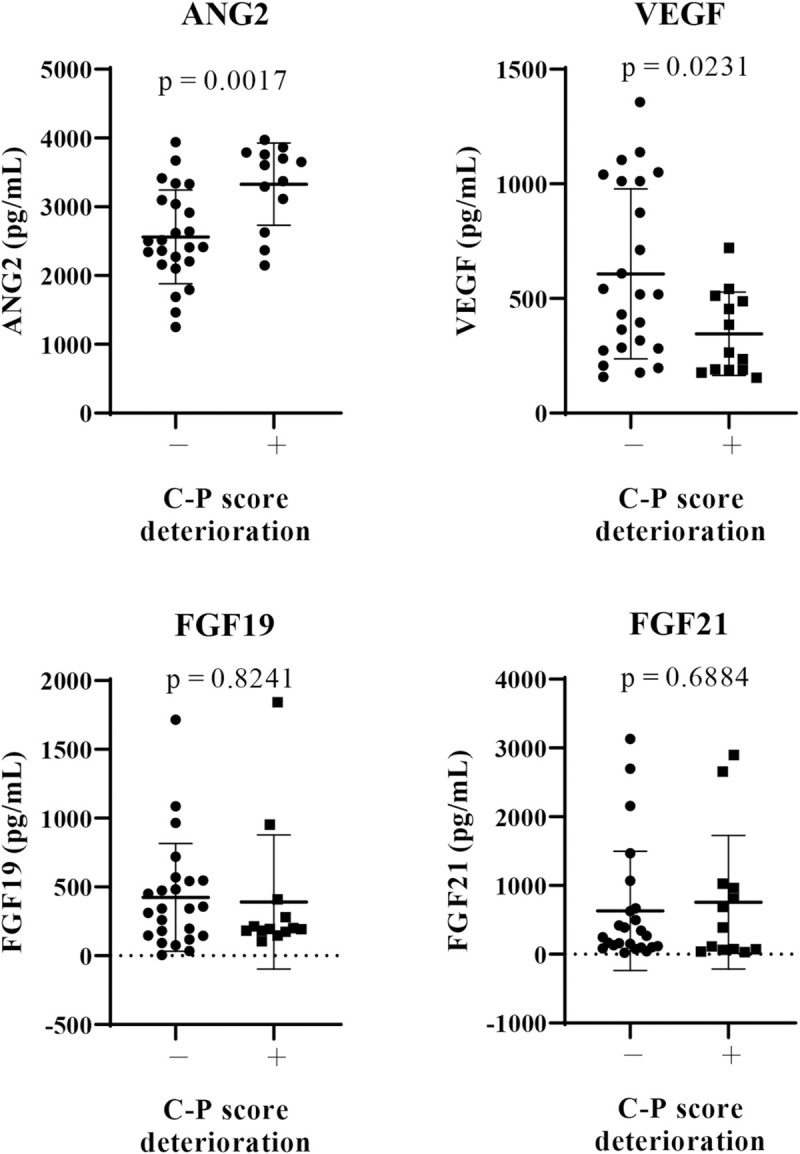
Comparison of baseline serum growth factors between patients with and without a deterioration of Child-Pugh score at 8 weeks after lenvatinib initiation. Baseline serum levels of FGF19, FGF21, ANG2, and VEGF were evaluated in all patients. We compared the mean values between patients with and those without deterioration of Child-Pugh (C-P) score at 8 weeks after lenvatinib initiation. *P* < 0.05 was considered statistically significant. FGF, fibroblast growth factor, ANG2: angiopoietin 2, VEGF: vascular endothelial growth factor.

**Fig 2:**
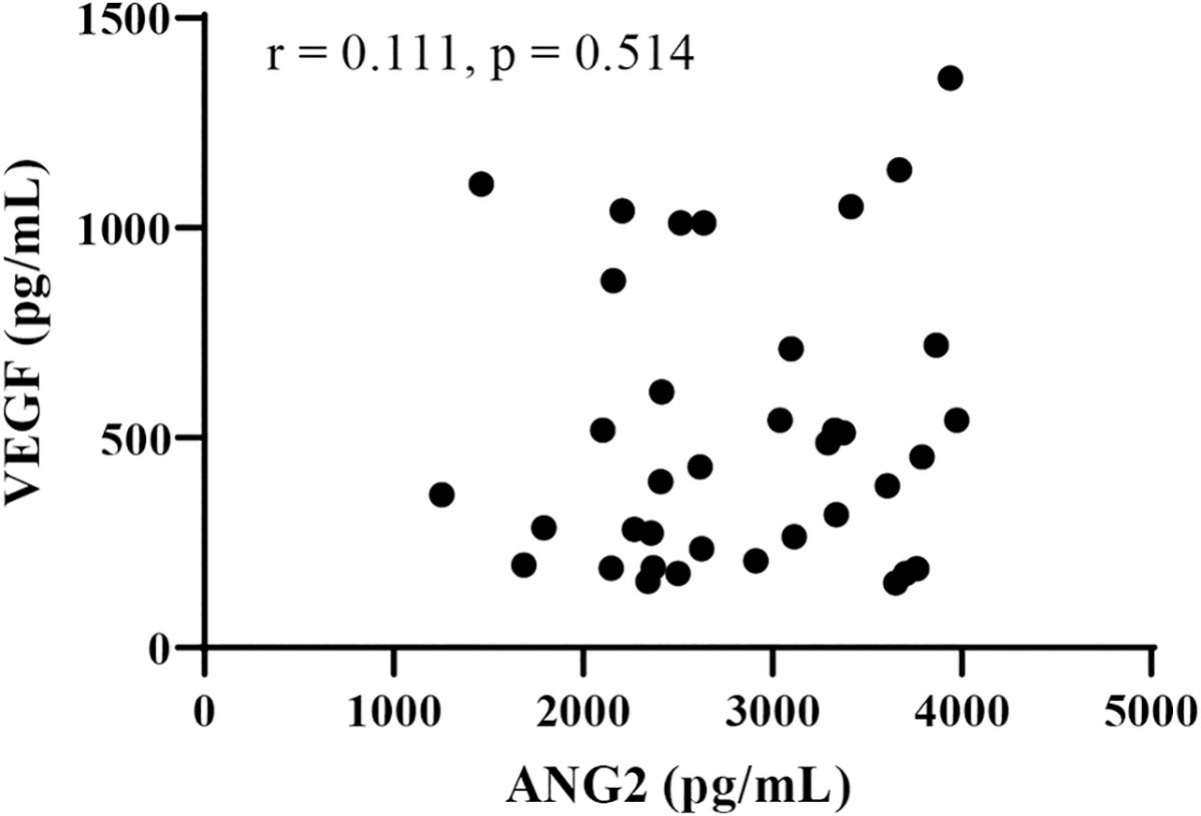
Correlation between baseline serum VEGF and ANG2 levels. ANG2: angiopoietin 2, VEGF: vascular endothelial growth factor.

Next, we analyzed the relationship between baseline serum growth factor levels (ANG2, VEGF, FGF19, and FGF21) and changes in the Child-Pugh score at 8 weeks after lenvatinib initiation. As shown in Fig 1, in patients with deteriorated Child-Pugh score after lenvatinib treatment, baseline ANG2 levels (*P* = 0.0017) were significantly higher and baseline VEGF levels (*P* = 0.0231) were significantly lower than those in patients without deteriorated Child-Pugh score. Baseline serum FGF19 and FGF21 levels were similar between these two patient groups. As shown in S3A Fig, baseline ANG2 levels were significant and positively correlated with the changes in the Child-Pugh score (ΔChild-Pugh score) (r = 0.4583, *P* = 0.043); meanwhile, baseline VEGF levels were significantly negatively correlated with theΔChild-Pugh score (r = -0.4181, *P* = 0.01). In addition, serum baseline ANG2 levels were significantly higher in patients with baseline Child-Pugh score of ≥7, compared with those with baseline Child-Pugh score of 5 (S3B Fig), and were significantly correlated with tumor size (r = 0.513, *P* = 0.004). While serum baseline VEGF levels were not associated with baseline Child-Pugh score and tumor factors (S3B Fig). No significant correlation was observed between baseline ANG2 and VEGF levels (r = 0.111, *P* = 0.514; Fig 2). In addition, multivariate analysis showed that both baseline ANG2 and VEGF levels were independent factors in predicting the deterioration of liver functional reserve (Table 4). Thus, we focused on baseline ANG2 and VEGF levels as predictors of deterioration of Child-Pugh score following lenvatinib.

**Table 4:** Multivariate analysis for factors associated with the deterioration of liver functional reserve during lenvatinib treatment.

	With deterioration	Without deterioration	Multivariate analysis	Odds ratio 95% (CI)
ANG2	3607 (2,150–3,972) ^a^ (pg/ML)	2,459 (1,253–3,940) ^a^ (pg/ML)	*P* = 0.004	1.003 (1.001–1.003)
VEGF	264 (154–720) ^a^ (pg/ML)	518 (158–1,356) ^a^ (pg/ML)	*P* = 0.025	0.993 (0.986–0.999)

ANG2: angiopoietin 2, VEGF: vascular endothelial growth factor, CI: confidence interval.

^a^ Data are shown as median (range) values.

### Classification of changes in Child-Pugh score based on combined baseline serum ANG2 and VEGF levels in patients treated with lenvatinib

**Fig 3:**
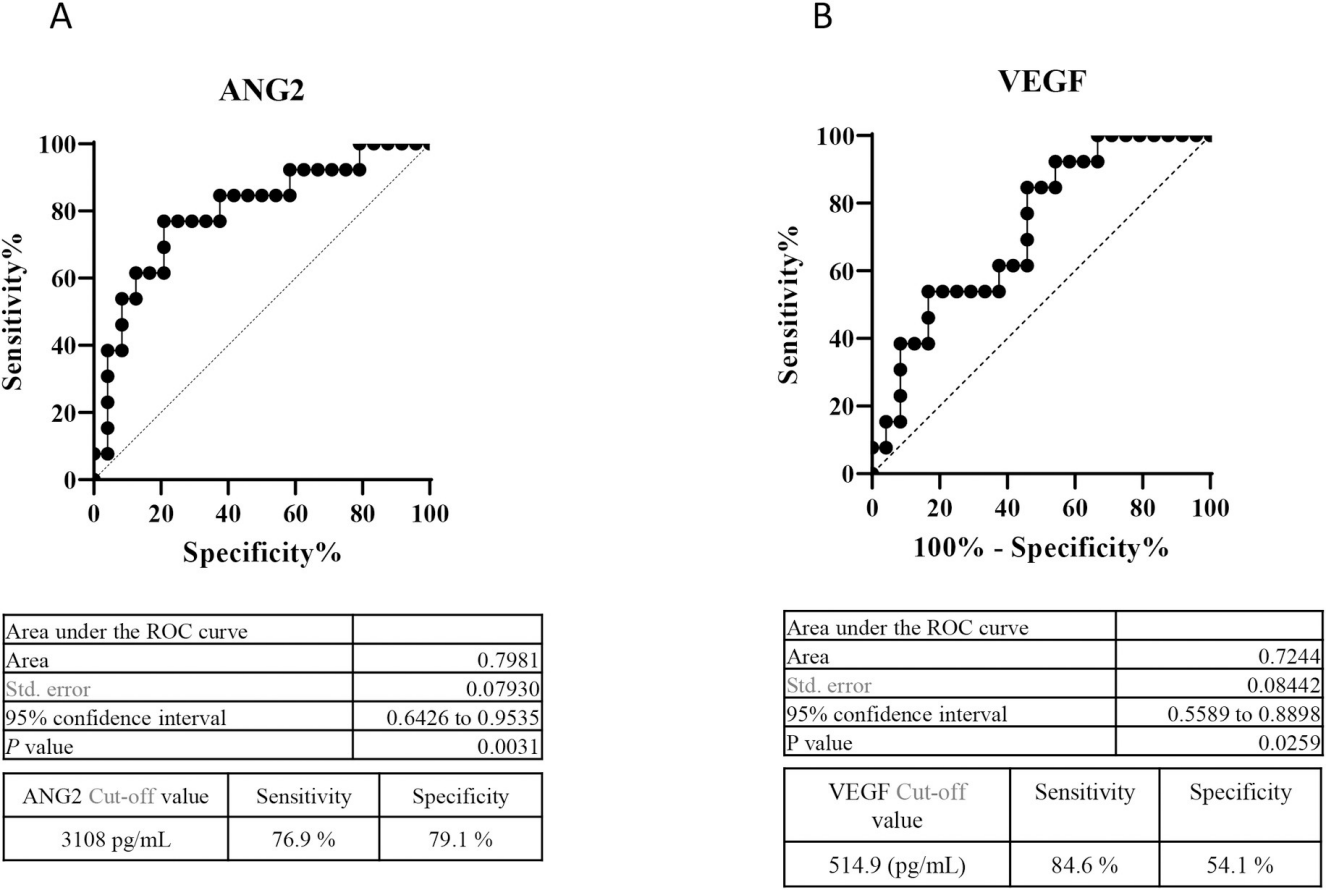
Cut-off values of baseline serum ANG2 and VEGF levels for predicting a reduction in the Child-Pugh score at 8 weeks after lenvatinib initiation. A. ROC curve analysis of baseline ANG2 levels in patients treated with lenvatinib. The cut-off baseline ANG2 level associated with a deteriorated Child-Pugh score at 8 weeks after lenvatinib initiation was 3,108 pg/mL (AUC = 0.772; sensitivity: 75.0%; specificity: 81.8%). B. ROC curve analysis of baseline VEGF levels in patients treated with lenvatinib. The cut-off baseline VEGF level associated with a deteriorated Child-Pugh score at 8 weeks after lenvatinib initiations was 194 pg/mL (AUC = 0.869; sensitivity: 75.0%; specificity: 81.8%).

As baseline high ANG2 and low VEGF levels were significantly associated with a deteriorated Child-Pugh score after lenvatinib for HCC, we conducted ROC analysis to determine cut-off values of baseline VEGF and ANG2 associated with a deteriorated Child-Pugh score at 8 weeks after lenvatinib initiation. As shown in Fig 3, the cut-off values for ANG2 and VEGF were 3,108 pg/mL (sensitivity: 0.769, specificity: 0.791, area under the ROC curve [AUC]: 0.7981, *P* = 0.0031) and 514.9 pg/mL (sensitivity: 0.846, specificity: 0.541, AUC: 0.7244, *P* = 0.0259), respectively.

**Fig 4:**
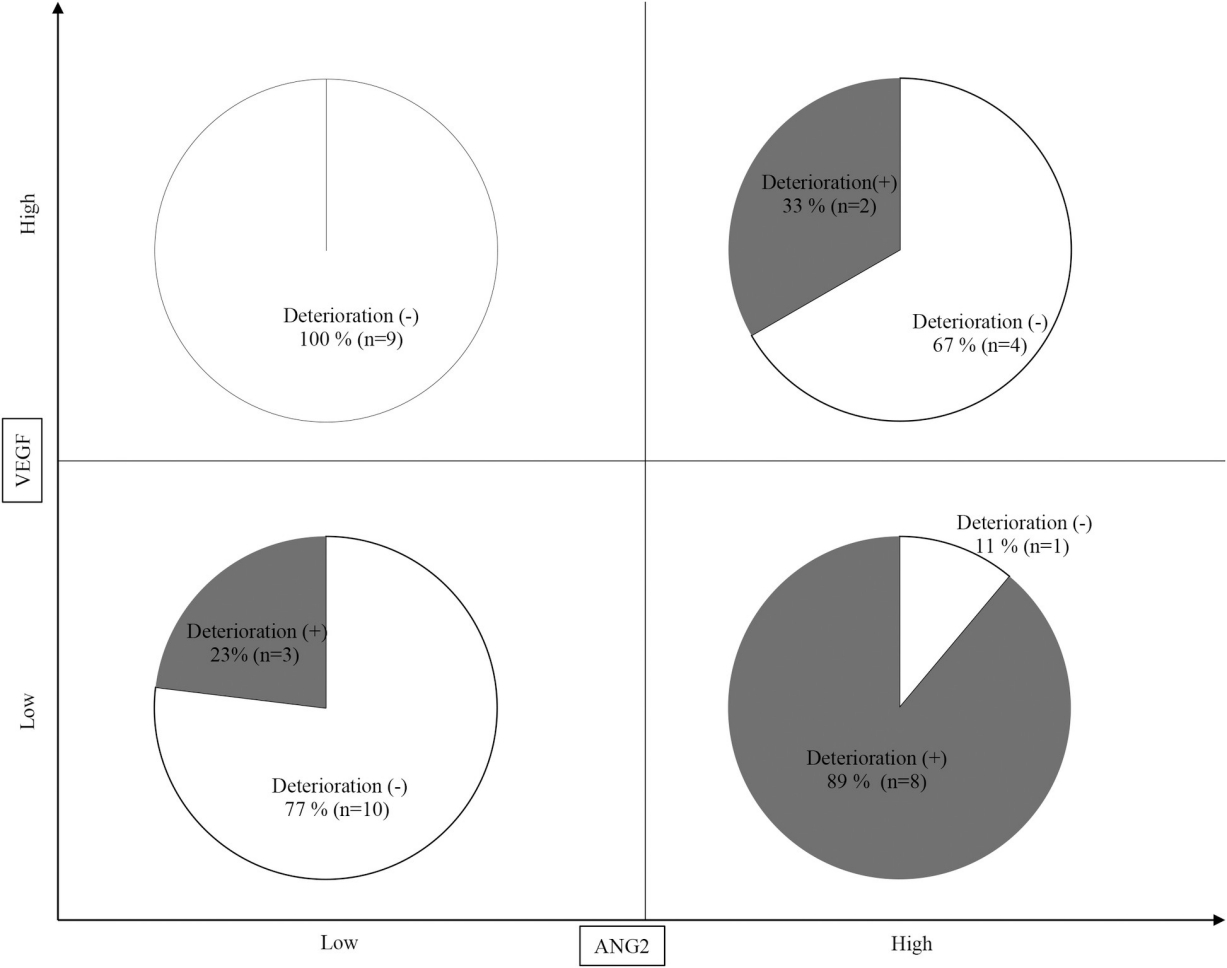
Prediction of deterioration of Child-Pugh score at 8 weeks after lenvatinib initiation based on baseline ANG2 and VEGF levels. Eight out of 9 (89%) patients with baseline low VEGF and high ANG2 levels showed a deterioration of Child-Pugh score at 8 weeks after lenvatinib initiation, whereas none of the patients with high VEGF and low ANG2 levels did.

Finally, we analyzed the changes in the Child-Pugh score after lenvatinib based on combined baseline ANG2 and VEGF levels. As shown in Fig 4, notably, 89% (8/9) of the patients with baseline low VEGF and high ANG2 levels showed a deteriorated Child-Pugh score, whereas none of the patient with baseline high VEGF and low ANG2 levels did. Thus, deterioration of Child-Pugh score in patients treated with lenvatinib for HCC may be predictable based on combined ANG2 and VEGF levels.

## Discussion

Liver functional reserve is one of the most important factors in determining therapeutic options in patients with HCC [21]. In clinical practice guidelines for HCC of The Japan Society of Hepatology [21], the Child-Pugh score, as an evaluation tool for liver functional reserves, is primarily considered to determine therapeutic options for HCC treatment. In consideration of post-progression TKI treatment, preserved liver functional reserve also is one of the most important factors. Recent developments in systemic chemotherapies for HCC have provided the option of sequential therapy regimens, which have the potential to prolong OS [22]; however, preserved liver functional reserve is a prerequisite for post-progression treatment. Importantly, Terashima et al. reported that a deterioration of Child-Pugh score during lenvatinib treatment for advanced HCC was an independent predictive factor for poor PFS and OS. Thus, prediction of the liver functional reserve response during TKI therapy is clinically important. In this study, although baseline general clinical factors, including HCC status and blood tests, could not predict a deterioration in the liver functional reserve during lenvatinib treatment for HCC, baseline high ANG2 and low VEGF serum levels were closely related to the deterioration of the liver functional reserve at 8 weeks after lenvatinib initiation.

A comparison of patients with Child-Pugh grades A and B showed that the Child-Pugh grade was not associated with the liver functional reserve after lenvatinib. However, among patients with a Child-Pugh score of 5, a total of 80% (12/15) maintained the score. This result is consistent with a recent study that reported that the introduction of lenvatinib in HCC patients with better hepatic function increased the chance of undergoing post-progression treatment [23].

ANG2 is implicated in the induction of vessel instability via an antagonistic effect on Tie2-mediated signaling [24, 25]. Therefore, high ANG2 levels are considered to be associated with vascular leak and inflammation. We recently reported that a high serum ANG2 level was associated with the existence of portal hypertension and non-regression of liver fibrosis, even after successful hepatitis C virus eradication by direct-acting antiviral agents [26]. Consistent with our results, it has been reported that portal hypertension-induced slow blood flow increases ANG2 expression [27, 28]. We and other groups have shown that, in patients with advanced HCC, high serum ANG2 levels were sometimes observed [29, 16]. Additionally, several recent studies have revealed that high ANG2 expression could predict pathological angiogenesis in nonalcoholic steatohepatitis, occurrence and recurrence of HCC after direct-acting antiviral therapy, mortality, and deterioration of kidney function in decompensated cirrhosis [28, 29, 30]. Collectively, these reports and the results of this study suggest that increased serum ANG2 levels might be associated with a deterioration of liver function via induction of vascular leak and inflammation.

We recently reported that baseline ANG2 levels were associated with treatment response to lenvatinib in patients with unresectable HCC [16]. Similarly, in this study, serum baseline ANG2 levels, not VEGF levels, were significantly associated with treatment response (S4 Fig). Thus, poor treatment response may be associated with the deterioration of the liver functional reserve. However, as shown in Table 2, treatment responses were similar between patients with and those without liver functional reserve deterioration. As ANG2 levels might be associated with liver functional reserve deterioration and treatment response, an evaluation of the baseline ANG2 level might be beneficial for patients with unresectable HCC for whom treatment with lenvatinib is planned.

As shown in Fig 2, ANG2 and VEGF levels were not correlated (r = 0.1108, *P* = 0.5139). Thus, the combination of baseline ANG2 and VEGF levels might be a useful biomarker to predict the deterioration of the liver functional reserve in patients with unresectable HCC who are treated with lenvatinib. Importantly, 89% (8/9) of the patients with baseline low VEGF and high ANG2 levels showed a deteriorated Child-Pugh-score at 8 weeks after lenvatinib initiation, whereas none of the patients (0/9) with baseline high VEGF and low ANG2 did.

Moritz et al. reported that in the absence of VEGF, ANG2 caused vessel destabilization and regression [31]. Thus, in case of low VEGF and high ANG2, the hepatic sinusoidal microvessel density might be reduced. Yang et al. reported that hepatic sinusoidal microvessel density was reduced by VEGF-specific blockade [19]. Thus, in patients with low hepatic sinusoidal microvessel density due to low VEGF and high ANG2, VEGF and FGF inhibitors might cause an additional decrease in hepatic blood flow, resulting in the deterioration of the liver functional reserve. Further basic and clinical investigations are required to evaluate this hypothesis.

This study had several limitations; it was a retrospective study, and the number of patients included was relatively limited. Therefore, further validation in future prospective studies with large sample sizes is required. Additionally, whether our findings are applicable to other systemic therapies for HCC remains to be investigated.

## Conclusions

Combined baseline serum ANG2 and VEGF levels may predict the deterioration of liver functional reserves in patients with unresectable HCC who are treated with lenvatinib.

## Supporting information

S1 Fig Study flow. (TIF)

S2 Fig The frequency of deteriorated Child-Pugh factors during the lenvatinib treatment. (TIF)

S3 Fig Association between baseline ANG2 and VEGF levels, changes in Child-Pugh score, and baseline Child-Pugh score. A. Correlation between baseline ANG2 or VEGF and changes in Child-Pugh score at 8 weeks post lenvatinib initiation. B. Comparison of baseline serum ANG2 and VEGF levels among patients with baseline Child-Pugh scores of 5, 6, and ≥7. ANG2: angiopoietin 2, VEGF: vascular endothelial growth factor,C-P score: Child-Pugh score, ΔChild-Pugh score: Changes in Child-Pugh score at 8 weeks post lenvatinib initiation.
(TIF)

S4 Fig Comparison of baseline serum ANG2 and VEGF levels between patients with or without object response. ANG2: angiopoietin 2, VEGF: vascular endothelial growth factor, CR: complete response, PR: partial response, SD: stable disease, PD: progressive disease.
(TIF)

## References

[1] Llovet JM, Burroughs A, Bruix J. Hepatocellular carcinoma. The Lancet. 2003;362(9399):1907–17. 10.1016/s0140-6736(03)14964-1 14667750

[2] Cheng AL, Kang YK, Lin DY, Park JW, Kudo M, Qin S, et al. Sunitinib versus sorafenib in advanced hepatocellular cancer: results of a randomized phase III trial. J Clin Oncol. 2013;31(32):4067–75. 10.1200/JCO.2012.45.8372 . 24081937

[3] Johnson PJ, Qin S, Park JW, Poon RT, Raoul JL, Philip PA, et al. Brivanib versus sorafenib as first-line therapy in patients with unresectable, advanced hepatocellular carcinoma: results from the randomized phase III BRISK-FL study. J Clin Oncol. 2013;31(28):3517–24. 10.1200/JCO.2012.48.4410 . 23980084

[4] Cainap C, Qin S, Huang WT, Chung IJ, Pan H, Cheng Y, et al. Linifanib versus Sorafenib in patients with advanced hepatocellular carcinoma: results of a randomized phase III trial. J Clin Oncol. 2015;33(2):172–9. 10.1200/JCO.2013.54.3298 25488963 PMC4279237

[5] Llovet JM, Ricci S, Mazzaferro V, Hilgard P, Gane E, Blanc JF, et al. Sorafenib in advanced hepatocellular carcinoma. N Engl J Med. 2008;359(4):378–90. 10.1056/NEJMoa0708857 . 18650514

[6] Bruix J, Qin S, Merle P, Granito A, Huang YH, Bodoky G, et al. Regorafenib for patients with hepatocellular carcinoma who progressed on sorafenib treatment (RESORCE): a randomised, double-blind, placebo-controlled, phase 3 trial. Lancet. 2017;389(10064):56–66. 10.1016/S0140-6736(16)32453-9 . 27932229

[7] Zhu AX, Kang YK, Yen CJ, Finn RS, Galle PR, Llovet JM, et al. Ramucirumab after sorafenib in patients with advanced hepatocellular carcinoma and increased alpha-fetoprotein concentrations (REACH-2): a randomised, double-blind, placebo-controlled, phase 3 trial. Lancet Oncol. 2019;20(2):282–96. 10.1016/S1470-2045(18)30937-9 . 30665869

[8] Kudo M, Finn RS, Qin S, Han KH, Ikeda K, Piscaglia F, et al. Lenvatinib versus sorafenib in first-line treatment of patients with unresectable hepatocellular carcinoma: a randomised phase 3 non-inferiority trial. Lancet. 2018;391(10126):1163–73. 10.1016/S0140-6736(18)30207-1 . 29433850

[9] Finn RS, Qin S, Ikeda M, Galle PR, Ducreux M, Kim TY, et al. Atezolizumab plus Bevacizumab in Unresectable Hepatocellular Carcinoma. N Engl J Med. 2020;382(20):1894–905. 10.1056/NEJMoa1915745 . 32402160

[10] D’Amico G, Garcia-Tsao G, Pagliaro L. Natural history and prognostic indicators of survival in cirrhosis: a systematic review of 118 studies. J Hepatol. 2006;44(1):217–31. 10.1016/j.jhep.2005.10.013 . 16298014

[11] Shigesawa T, Maehara O, Suda G, Natsuizaka M, Kimura M, Shimazaki T, et al. Lenvatinib suppresses cancer stem-like cells in HCC by inhibiting FGFR 1–3 signaling, but not FGFR4 signaling. Carcinogenesis. 2020. 10.1093/carcin/bgaa049 . 32449510

[12] Hiraoka A, Kumada T, Kariyama K, Takaguchi K, Itobayashi E, Shimada N, et al. Therapeutic potential of lenvatinib for unresectable hepatocellular carcinoma in clinical practice: Multicenter analysis. Hepatol Res. 2019;49(1):111–7. 10.1111/hepr.13243 . 30144256

[13] Maruta S, Ogasawara S, Ooka Y, Obu M, Inoue M, Itokawa N, et al. Potential of Lenvatinib for an Expanded Indication from the REFLECT Trial in Patients with Advanced Hepatocellular Carcinoma. Liver Cancer. 2020;9(4):382–96. 10.1159/000507022 32999866 PMC7506220

[14] Shigesawa T, Suda G, Kimura M, Shimazaki T, Maehara O, Yamada R, et al. Baseline angiopoietin-2 and FGF19 levels predict treatment response in patients receiving multikinase inhibitors for hepatocellular carcinoma. JGH Open. 2020;4(5):880–8. 10.1002/jgh3.12339 33102759 PMC7578287

[15] Kawamura Y, Kobayashi M, Shindoh J, Kobayashi Y, Kasuya K, Sano T, et al. (18)F-Fluorodeoxyglucose Uptake in Hepatocellular Carcinoma as a Useful Predictor of an Extremely Rapid Response to Lenvatinib. Liver Cancer. 2020;9(1):84–92. 10.1159/000503577 32071912 PMC7024860

[16] Sho T, Suda G, Ogawa K, Kimura M, Shimazaki T, Maehara O, et al. Early response and safety of lenvatinib for patients with advanced hepatocellular carcinoma in a real-world setting. JGH Open. 2020;4(1):54–60. 10.1002/jgh3.12209 32055698 PMC7008153

[17] Sho T, Suda G, Ogawa K, Shigesawa T, Suzuki K, Nakamura A, et al. Lenvatinib in patients with unresectable hepatocellular carcinoma who do not meet the REFLECT trial eligibility criteria. Hepatol Res. 2020;50(8):966–77. 10.1111/hepr.13511 . 32562334

[18] Terashima T, Yamashita T, Takata N, Toyama T, Shimakami T, Takatori H, et al. Comparative analysis of liver functional reserve during lenvatinib and sorafenib for advanced hepatocellular carcinoma. Hepatol Res. 2020;50(7):871–84. 10.1111/hepr.13505 . 32307874

[19] Yang Y, Zhang Y, Cao Z, Ji H, Yang X, Iwamoto H, et al. Anti-VEGF- and anti-VEGF receptor-induced vascular alteration in mouse healthy tissues. Proc Natl Acad Sci U S A. 2013;110(29):12018–23. 10.1073/pnas.1301331110 23818623 PMC3718173

[20] Lencioni R, Llovet JM. Modified RECIST (mRECIST) assessment for hepatocellular carcinoma. Seminars in liver disease. 2010;30(1):52–60. 10.1055/s-0030-1247132 . 20175033 PMC12268942

[21] Kokudo N, Takemura N, Hasegawa K, Takayama T, Kubo S, Shimada M, et al. Clinical practice guidelines for hepatocellular carcinoma: The Japan Society of Hepatology 2017 (4th JSH-HCC guidelines) 2019 update. Hepatol Res. 2019;49(10):1109–13. 10.1111/hepr.13411 . 31336394

[22] Ogasawara S, Ooka Y, Itokawa N, Inoue M, Okabe S, Seki A, et al. Sequential therapy with sorafenib and regorafenib for advanced hepatocellular carcinoma: a multicenter retrospective study in Japan. Invest New Drugs. 2020;38(1):172–80. 10.1007/s10637-019-00801-8 . 31172442

[23] Hiraoka A, Kumada T, Fukunishi S, Atsukawa M, Hirooka M, Tsuji K, et al. Post-Progression Treatment Eligibility of Unresectable Hepatocellular Carcinoma Patients Treated with Lenvatinib. Liver Cancer. 2020;9(1):73–83. 10.1159/000503031 32071911 PMC7024896

[24] Souma T, Thomson BR, Heinen S, Carota IA, Yamaguchi S, Onay T, et al. Context-dependent functions of angiopoietin 2 are determined by the endothelial phosphatase VEPTP. Proc Natl Acad Sci U S A. 2018;115(6):1298–303. 10.1073/pnas.1714446115 29358379 PMC5819405

[25] Benest AV, Kruse K, Savant S, Thomas M, Laib AM, Loos EK, et al. Angiopoietin-2 is critical for cytokine-induced vascular leakage. PLoS One. 2013;8(8):e70459. 10.1371/journal.pone.0070459 23940579 PMC3734283

[26] Kawagishi N, Suda G, Kimura M, Maehara O, Shimazaki T, Yamada R, et al. High serum angiopoietin-2 level predicts non-regression of liver stiffness measurement-based liver fibrosis stage after direct-acting antiviral therapy for hepatitis C. Hepatol Res. 2020;50(6):671–81. 10.1111/hepr.13490 . 32020702

[27] Goettsch W, Gryczka C, Korff T, Ernst E, Goettsch C, Seebach J, et al. Flow-dependent regulation of angiopoietin-2. J Cell Physiol. 2008;214(2):491–503. 10.1002/jcp.21229 . 17960565

[28] Faillaci F, Marzi L, Critelli R, Milosa F, Schepis F, Turola E, et al. Liver Angiopoietin-2 Is a Key Predictor of De Novo or Recurrent Hepatocellular Cancer After Hepatitis C Virus Direct-Acting Antivirals. Hepatology. 2018;68(3):1010–24. 10.1002/hep.29911 29604220 PMC6175123

[29] Allegretti AS, Vela Parada X, Ortiz GA, Long J, Krinsky S, Zhao S, et al. Serum Angiopoietin-2 Predicts Mortality and Kidney Outcomes in Decompensated Cirrhosis. Hepatology. 2019;69(2):729–41. 10.1002/hep.30230 30141205 PMC6351209

[30] Lefere S, Van de Velde F, Hoorens A, Raevens S, Van Campenhout S, Vandierendonck A, et al. Angiopoietin-2 Promotes Pathological Angiogenesis and Is a Therapeutic Target in Murine Nonalcoholic Fatty Liver Disease. Hepatology. 2019;69(3):1087–104. 10.1002/hep.30294 . 30259536

[31] Moritz F, Schniering J, Distler JHW, Gay RE, Gay S, Distler O, et al. Tie2 as a novel key factor of microangiopathy in systemic sclerosis. Arthritis Res Ther. 2017;19(1):105. 10.1186/s13075-017-1304-2 28545512 PMC5445339

