# Supplementary figures and images for: Baseline serum angiopoietin-2 and VEGF levels predict the deterioration of the liver functional reserve during lenvatinib treatment for hepatocellular carcinoma
Source: PLoS One. 2021 Mar 1;16(3):e0247728. doi: 10.1371/journal.pone.0247728 (PMC7920365; doi:10.1371/journal.pone.0247728)

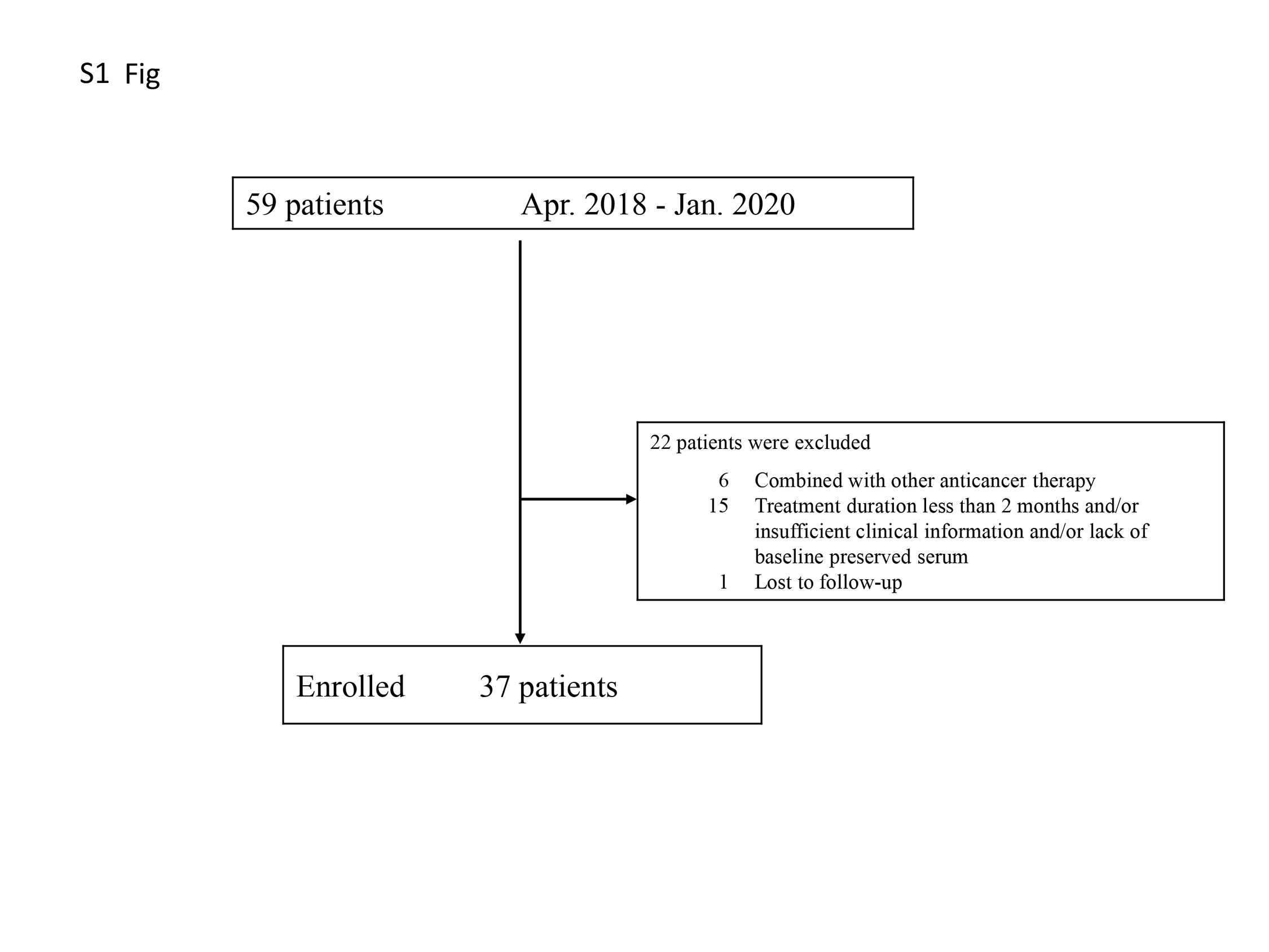

Supplement: S1 Fig — (TIF) [file pone.0247728.s001.tif]

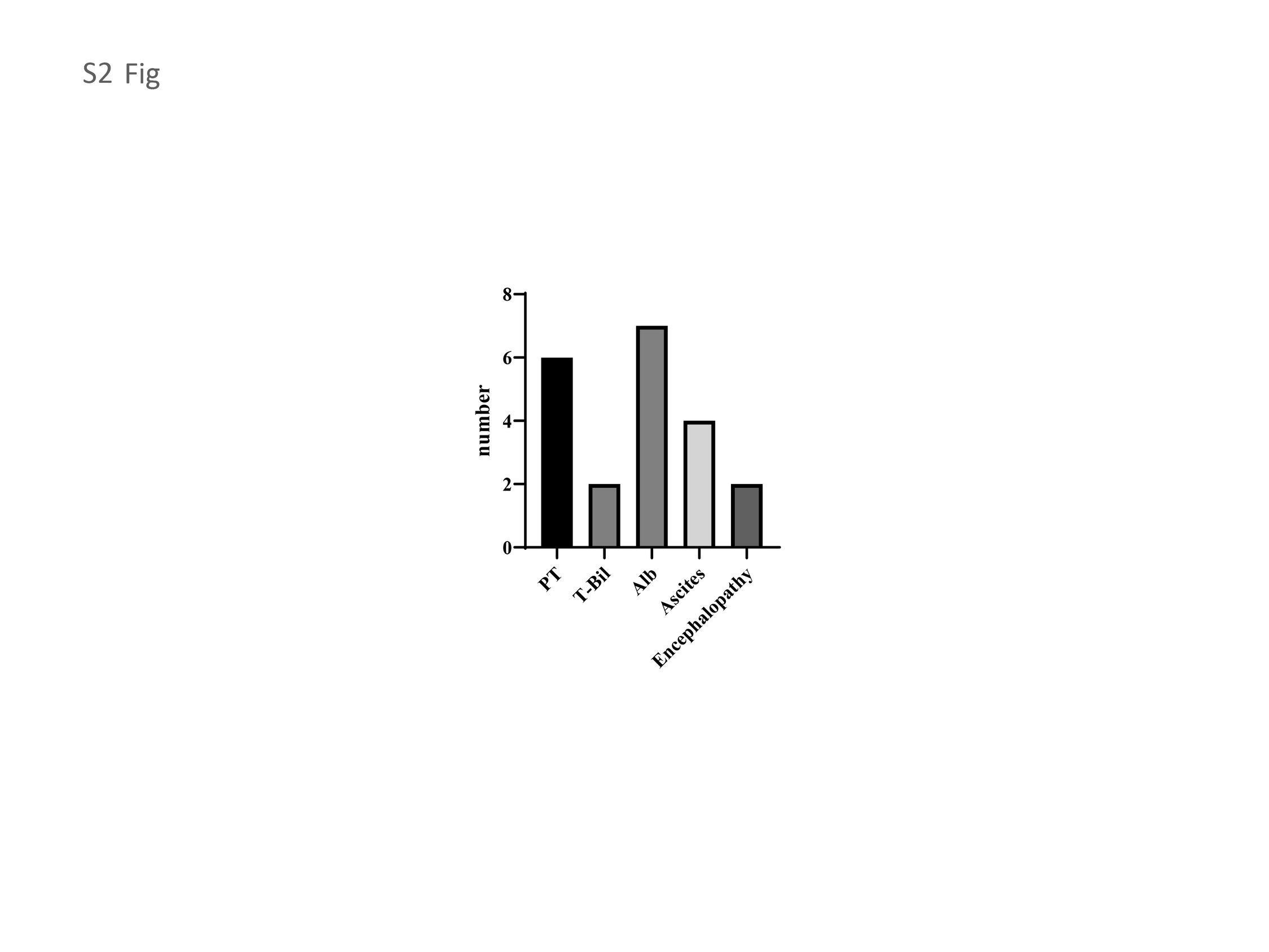

Supplement: S2 Fig — (TIF) [file pone.0247728.s002.tif]

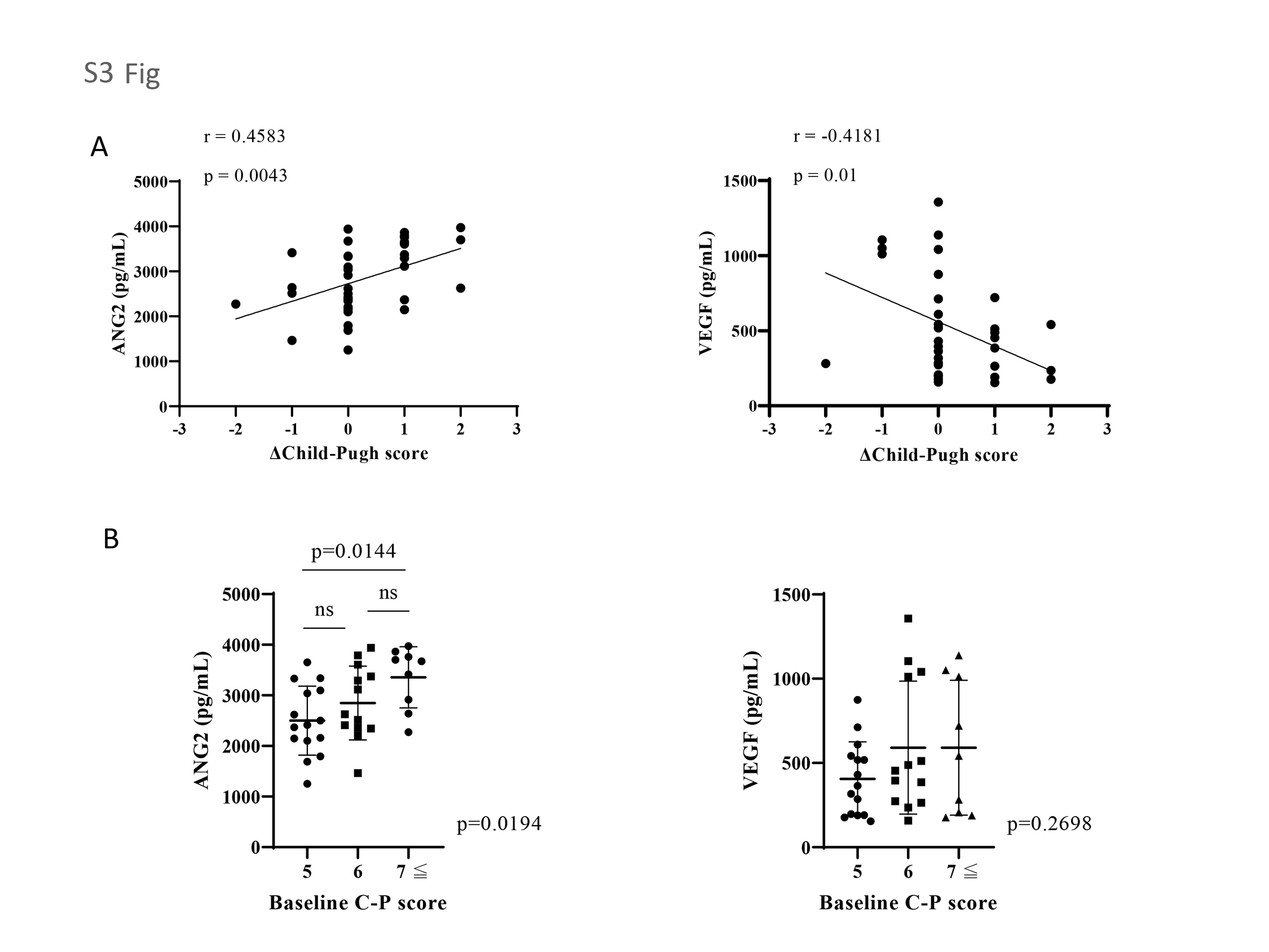

Supplement: S3 Fig — A. Correlation between baseline ANG2 or VEGF and changes in Child-Pugh score at 8 weeks post lenvatinib initiation. B. Comparison of baseline serum ANG2 and VEGF levels among patients with baseline Child-Pugh scores of 5, 6, and ≥7. ANG2: angiopoietin 2, VEGF: vascular endothelial growth factor,C-P score: Child-Pugh score, ΔChild-Pugh score: Changes in Child-Pugh score at 8 weeks post lenvatinib initiation. (TIF) [file pone.0247728.s003.tif]

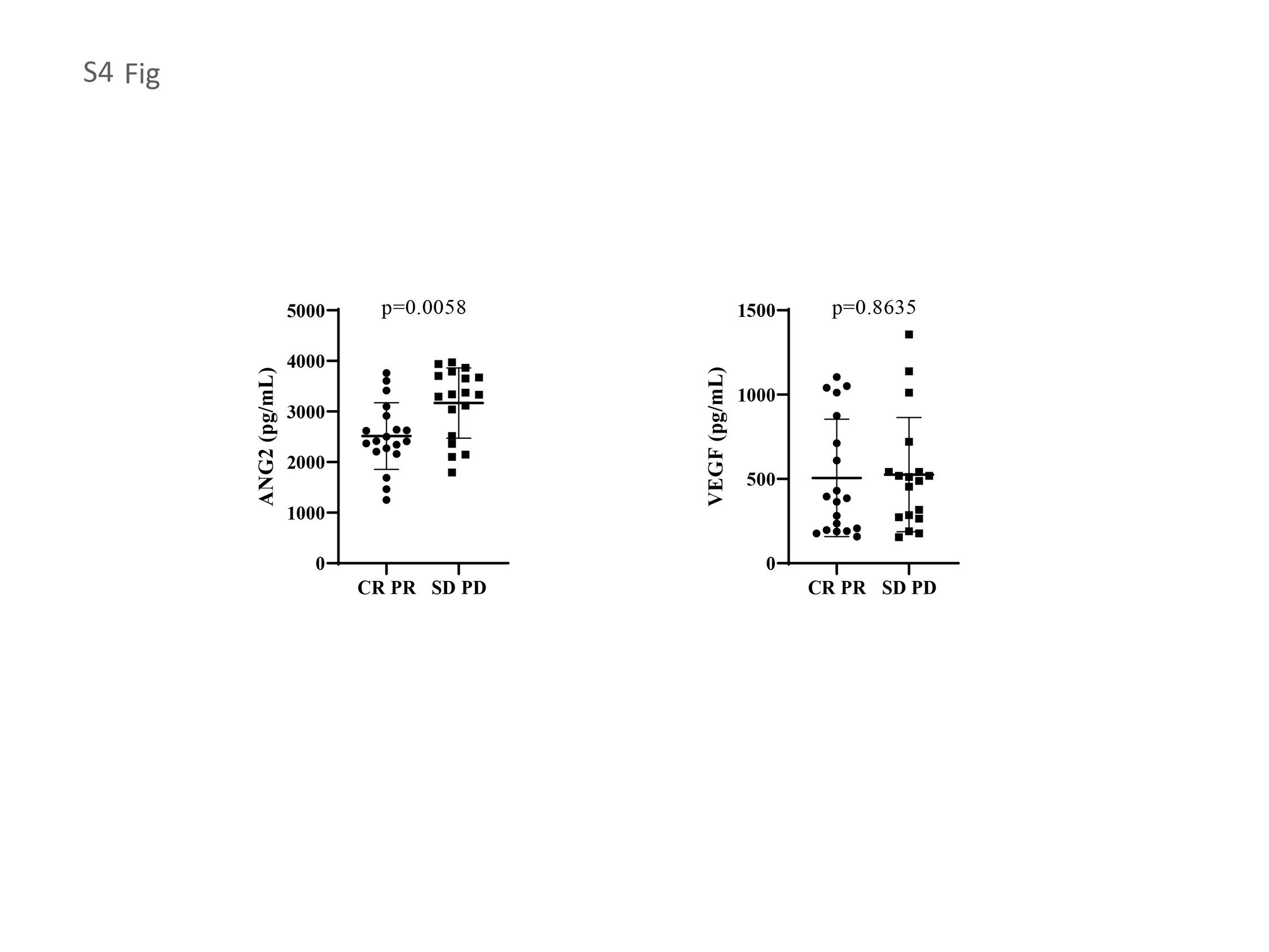

Supplement: S4 Fig — ANG2: angiopoietin 2, VEGF: vascular endothelial growth factor, CR: complete response, PR: partial response, SD: stable disease, PD: progressive disease. (TIF) [file pone.0247728.s004.tif]
